# Modified protocol of omalizumab treatment to prevent carboplatin-induced drug hypersensitivity reactions: a case study

**DOI:** 10.1186/s13601-020-0309-0

**Published:** 2020-01-29

**Authors:** Hanneke N. G. Oude Elberink, Mathilde Jalving, Hilda Dijkstra, Annick A. J. M. van de Ven

**Affiliations:** 1 Department of Allergology and Internal Medicine, Internal address code AA21, University Medical Center Groningen, Hanzeplein 1, 9713 GZ Groningen, The Netherlands; 2 Department of Medical Oncology, University Medical Center Groningen, Groningen, The Netherlands; 3 Department of Clinical Pharmacy and Pharmacology, University Medical Center Groningen, Groningen, The Netherlands

**Keywords:** Carboplatin, Desensitisation, Drug hypersensitivity, Omalizumab

## Abstract

Carboplatin administration can usually be safely continued via a so-called desensitisation protocol when hypersensitivity reactions arise. Severe break-through reactions that occur early during desensitisation are likely to be IgE-mediated; in that case, addition of omalizumab premedication should be strongly considered.

## To the editor

Platinum-based chemotherapy is the cornerstone in the treatment of various solid tumours, including gynaecologic malignancies. The incidence of drug hypersensitivity reactions (DHRs) is high; up to 12% for carboplatin in gynaecological tumours [1]. The pathogenesis of platin-related DHRs may vary but for carboplatin, IgE-specific basophil activation has been demonstrated [2].

Fortunately, patients with a DHR to carboplatin can generally still be safely treated with carboplatin using a desensitisation protocol [3]. Protocols rely on two main principles, namely gradually increasing the dose of drug and using a premedication consisting of a combination of H1-, H2-antihistamines, corticosteroids and in some cases a leukotriene antagonist [3]. This method is successful for most patients; however, some still suffer from symptoms despite intense pre-treatment and extra anti-allergy medication during the desensitisation procedure. We describe a patient who developed a systemic allergic reaction at the first step (1 mg carboplatin/hour) of the desensitisation schedule on two separate occasions. Carboplatin treatment could, however, be successfully continued after pre-treatment with omalizumab and no further adverse events occurred.

**Fig. 1:**
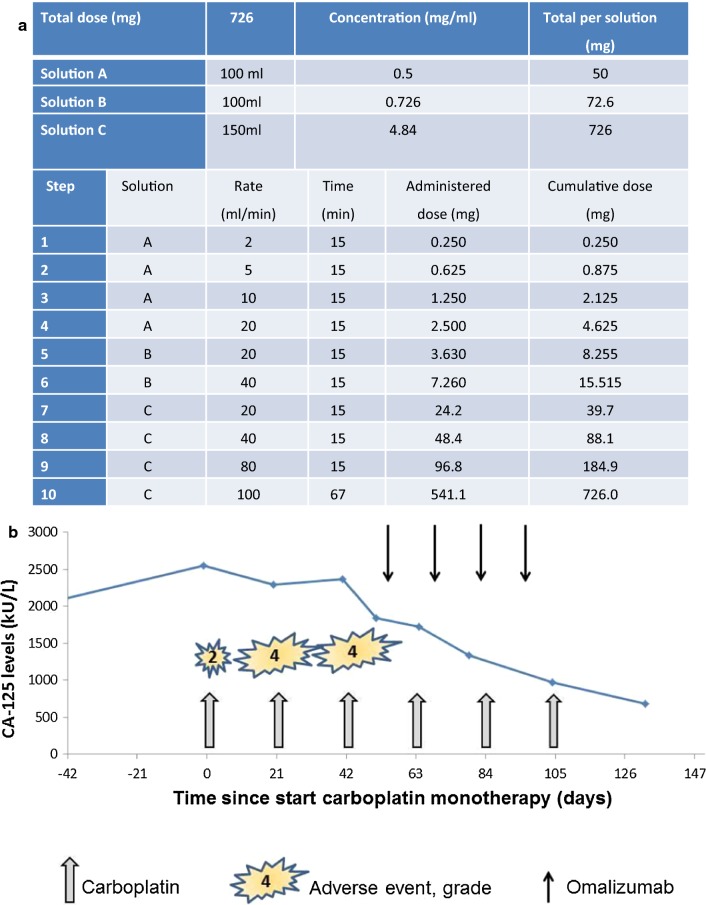
Management of carboplatin allergy. **a** 10-step desensitisation schedule for carboplatin. Cumulative dose as administered in the 6th and last cycle of the course. **b** Overview of carboplatin and omalizumab administration in relation to the adverse allergic reactions

The case concerns a now 57-year-old woman diagnosed with stage III ovarian cancer of the endometrioid type in 2008 (Table 1). In 2014, she had a platinum-sensitive relapse without rational surgical options and palliative chemotherapy with carboplatin/paclitaxel was initiated. During the second cycle, she developed an allergic reaction consisting of patchy erythema, coughing, throat and chest discomfort. The chemotherapy was stopped and referral to an allergologist followed. Carboplatin hypersensitivity was diagnosed based on the clinical presentation in combination with skin tests positive for carboplatin (Table 2). Three subsequent cycles of carboplatin were given according to a 10-step desensitisation schedule and were uneventful. (Figure 1a).

**Table 1:** Summary of clinical events and treatment over time

Year	Event	Surgical debulking	Adjuvant chemotherapy	Allergology
2008	Stage III ovarian cancer of the endometrioid type	Yes	6 Cycles of carboplatin and paclitaxel	
2009				
2010	Disease relapse	Yes	No	
2011				
2012				
2013	Disease relapse	Yes	No	
2014	Symptomatic platinum-sensitive disease relapse	Not possible	2 Cycles of carboplatin/paclitaxel Cycle 3 omitted Cycle 4–6 according to 10-step desensitisation schedule	Cycle 2: allergic reaction Skin tests positive for carboplatin, negative for paclitaxel
2015				
2016				
2017	Symptomatic platinum-sensitive disease relapse	No	6 Cycles of carboplatin/paclitaxel according to 10-step desensitisation schedule	Skin tests positive for carboplatin
2018	Symptomatic platinum-sensitive disease relapse	No	6 Cycles of carboplatin monotherapy according to 10-step desensitisation schedule with additional omalizumab for cycle 4–6	Cycle 1: flushing, pruritus and erythema of the face and chest Cycle 2 + 3: Anaphylaxis Skin tests positive for carboplatin (negative for cisplatin) Cycle 4–6: Uneventful Skin tests persistently positive
2019	Symptomatic platinum-sensitive disease relapse	No	6 Cycles of carboplatin monotherapy according to 10-step desensitisation schedule with additional omalizumab	No events

**Table 2:** Diagnostic testing in suspected carboplatin allergy

Time after initial diagnosis (years)	6.5	9.5	11	11.2
Status	Prior to 2nd series of carboplatin/paclitaxel	Prior to 3rd series of carboplatin/paclitaxel	After 3 cycles carboplatin monotherapy (4th series), 0x omalizumab	After 6 cycles carboplatin (4th series) and 4x omalizumab*
Saline, diameter (mm)	0	0	0	0
Histamine, diameter (mm)	9.5	4	7.5	6
Drugs tested: diameter of wheal in mm				
Carboplatin 0.01 mg/ml	*8.5*	0	*6*	0
Carboplatin 0.1 mg/ml	N/A	0	*7*	*6.5*
Carboplatin 1 mg/ml	N/A	*4.5*	*7.5*	*5.5*
Paclitaxel 0.001 mg/ml	0	N/A	N/A	N/A
Paclitaxel 0.01 mg/ml	0	N/A	N/A	N/A
Paclitaxel 0.1 mg/ml	0	N/A	N/A	N/A
Paclitaxel 1 mg/ml	0	N/A	N/A	N/A
Cisplatin 0.01 mg/ml	N/A	N/A	0	0
Cisplatin 0.1 mg/ml	N/A	N/A	0	0
Cisplatin 1 mg/ml	N/A	N/A	0	0

Overview of intracutaneous testing for carboplatin and other chemotherapeutics. Positive results are shown in italics. Diameter = average of the length and width of the wheal, read 15–20 min after intracutaneous injection of the drug

*N/A* not assessed

* Skin tests were performed 8 weeks after the last omalizumab injection

The desensitisation procedure was successfully repeated with a relapse 3 years later. In 2018, carboplatin monotherapy was initiated due to a third symptomatic platinum-sensitive relapse. During the first cycle, an allergic reaction occurred at the last desensitisation step (Fig. 1b). The reaction consisted of flushing, pruritus and erythema of the face and chest. The carboplatin infused was stopped and intravenous clemastine was administered resulting in resolution of symptoms within minutes. The desensitisation was successfully continued at the penultimate infusion rate. 3 weeks later she experienced a more severe reaction moments after commencing the first infusion step, despite pre-treatment with H1/H2-antihistamines and dexamethasone. She had symptoms of flushing, hypotension, dyspnoea with chest discomfort, throat tightness and abdominal discomfort. Additional administration of clemastine, ranitidine and dexamethasone had insufficient effect and 0.5 mg of intramuscular epinephrine was required to relieve symptoms. There was no alternative explanation for this reaction, i.e. no co-factors such as concurrent infection, recent exercise or use of novel medications. After administration of the abovementioned medication, the desensitisation could be continued according to protocol without further additional medication or adverse events. During administration of the third cycle, despite optimizing premedication (20 mg dexamethasone i.v., 50 mg ranitidine i.v., 2 mg clemastine i.v. and 10 mg montelukast orally, all ≥ 1 h prior to the first infusion), a similar anaphylactic reaction occurred at the first infusion step. Intramuscular epinephrine halted the allergic reaction and again, the desensitisation could be completed without other events.

Since further dilution of the carboplatin to allow an even slower desensitisation was not possible (in accordance to the SmPC of Carboplatin), other potential solutions were explored. Ojaimi et al. [4] described a patient who failed their 2-day and subsequently 4-day desensitisation protocol for carboplatin. After 3 fortnightly doses of 300 mg of omalizumab, a monoclonal anti-IgE antibody, carboplatin was successfully administered over 4 days.

We opted to aim to reduce the burden of anti-carboplatin IgE-antibodies by administering omalizumab. Our patient received one dose of omalizumab 300 mg 2 weeks before the 4th cycle of carboplatin was administered, and continued fortnightly (Fig. 1b). The following three administrations of carboplatin occurred without any side effects and no adaptations to the desensitisation protocol were required. Omalizumab was well tolerated. She had a good clinical and partial radiological response to the chemotherapy with 73% decreased CA-125 titres and commenced maintenance treatment with niraparib 6 weeks after the last cycle of chemotherapy. Unfortunately, she relapsed within 6 months and carboplatin monotherapy was reinitiated. The anti-allergy premedication regimen included omalizumab 300 mg every 14 days (first injection was given 11 days prior to the first cycle) and the desensitisation procedure was carried out uneventfully.

We here describe the successful addition of omalizumab to the conventional anti-allergic medication in a patient with severe break-through allergic reactions to carboplatin despite an optimized desensitisation schedule. To our knowledge, this is the second time omalizumab has been used as an adjuvant during carboplatin desensitisation. Ojaimi and colleagues added omalizumab to a more conservative desensitisation protocol. Our results confirm their findings and suggest that one dose of omalizumab prior to the start of desensitisation may already be sufficient, thereby minimizing treatment delay and enabling desensitisation procedures to be kept at the regular time schedule of 3.5 h.

There is limited but growing experience using omalizumab for desensitisation of DHR; case-reports or small case series describe positive results for aspirin [5], insulin [6], Elosulfase A, [7] and recently oxaliplatin [8]. Careful selection of patients remains pivotal and sufficient knowledge regarding the underlying pathogenic mechanism of the allergic reaction is essential. Non-IgE-mediated reactions are less likely to fully respond to this therapy. Consequently, the mechanism of hypersensitivity reactions should ideally be substantiated by diagnostics in order to identify those patients that might benefit from the addition of omalizumab. Carboplatin-induced DHR are IgE-mediated, as specific anti-carboplatin IgE antibodies can be detected in patients with DHR to carboplatin [9]. Iwamoto et al. nicely demonstrated in vitro an IgE-dependent mechanism in patients with carboplatin DHR [2]. The carboplatin reactivity was transferable when plasma of these patients was added to healthy control basophils, but could be almost completely blocked when cells were pre-treated with omalizumab.

For our patient, measurement of anti-carboplatin IgE was not available and a basophil activation test was unsuccessful probably due to the presence of low levels of omalizumab in the sample. Skin tests however repeatedly showed reactivity to carboplatin, which supports the presence of an IgE-mediated DHR.

Taken together, for patients with continued allergic reactions of established or strongly suspected IgE-mediated origin despite a desensitisation schedule including conventional anti-allergic medication, we recommend additional pre-treatment with one dose of omalizumab 1–3 weeks prior to each cycle of chemotherapy.

In conclusion, omalizumab can be a valuable addition to the allergologist’s repertoire for desensitisation in case of patients suffering from adverse reactions suggestive of an IgE-mediated allergy.

## Data Availability

The datasets used and analysed during the current study are available from the corresponding author on reasonable request.

## Abbreviation

DHR: drug hypersensitivity reaction
